# Prevalence of Plastic Usage and the Factors Associated With It Among Adults in Perambalur District of South India: A Cross-Sectional Study

**DOI:** 10.7759/cureus.46294

**Published:** 2023-09-30

**Authors:** Karthikeyan Duraisamy, Tamilarasan Muniyapillai, Karthikeyan Kulothungan, Pavithra Mahendran, Ramakalam Ayyappan, Ramanan Rengaraj, Ramya Senthil Velan, Rasika Muralitharan, Ramachandiran Nagarajan, Reshika Manohar

**Affiliations:** 1 General Surgery, Government Medical College, Tiruppur, Tiruppur, IND; 2 Community Medicine, Dhanalakshmi Srinivasan Medical College and Hospital, Perambalur, IND

**Keywords:** waste management, plastic ban, plastic alternatives, plastic hazards, plastic usage, plastics

## Abstract

Background

People extensively use and dispose of plastic products because of their durability, affordability, and lightweight. The excessive production and consumption of plastics has led to pollution that has negative effects on both society and the environment. Nearly all Indian states and union territories have prohibited the usage of plastic bags, with Tamil Nadu being the fourth state in the country to implement such a prohibition. The study aims to determine the prevalence of plastic usage, its associated factors, and awareness regarding the laws related to the plastic ban.

Methodology

We conducted a cross-sectional study on 1200 adults above 18 years of age using multistage sampling. We undertook the study for three months in the district of Perambalur, in South India. The semi-structured questionnaire was used as a research tool, which contained information on socio-demographics, plastic utilization, understanding of plastic prohibition, its need, and views on bisphenol A (BPA). We entered the gathered data in Microsoft Excel (Microsoft Corporation, Redmond, WA) and analyzed using SPSS version 26 (IBM Corp., Armonk, NY). If the P-value was less than 0.05, we deemed it statistically significant.

Results

The mean age of the study participants was 44.47 ± 15.09 years. Plastic is being used by most of the study participants because of its wide availability and convenience of usage. Approximately 42.43% (n=471) of the participants adhere to non-segregation waste disposal practices in public bins. Approximately 1100 (92.5%) of the participants incorporate plastic into their daily routine. Among the participants, approximately 15.7% (188) were knowledgeable about bisphenol A (BPA), while about 92.6% (1111) of them were knowledgeable about plastic substitutes. Individuals belonging to the younger age group, male gender, higher education background, living in urban areas, living in joint families, and not engaged in agriculture were the primary users of plastic, and this trend was statistically significant (p <0.001).

Conclusion

In the study, the prevalence of plastic usage was higher among the participants who followed unsanitary methods of disposal. Despite the awareness of the hazards of plastic and the regulations against its use, its consumption remains high. Plastic consumption is higher in the urbanized area across residential, educational, occupational, and young age demographics. The mere act of raising awareness is insufficient; it is necessary to convert awareness into action to protect both the environment and humanity.

## Introduction

Plastics, owing to their ease of use and affordability, have taken over every aspect of our modern world [1]. It has become an essential aspect of our everyday lives, permeating every sector from food packaging to toys and electronics, to the degree that it is unimaginable to picture a world without it. Plastic offers undeniable benefits, including its versatility, cost-effectiveness, lightweight nature, resistance to degradation, and reduced energy requirements for production [2,3]. Plastic not only has these advantages but also has some disadvantages, such as improper disposal, as plastic takes around 15 to 1000 years to biodegrade [4]. The impact of fuel-based plastics on the industrial world has been revolutionary, with every area of manufacturing being touched by their versatility [5].

Exposure to toxic chemicals found in plastic, such as bisphenol A (BPA), poly-halogenated compounds, styrene, phthalates, etc., can cause diabetes, cardiovascular disease, and liver illness [4]. The adverse health effects associated with plastic use are many and include dysgenesis, cryptorchidism, infertility, obesity, polycystic ovarian disease in women, and even cancer in animal studies [1,6]. Besides the aforementioned problems, the choking of drains because of plastic waste not only serves as a breeding site for vectors but also contributes to the spread of malaria in endemic areas [4].

Single-use plastic refers to a plastic item that is intentionally designed to be used only once and discarded. The consumption of plastic by individuals and the ineffective reuse or recycling of it has detrimental effects on the ecosystem, leading to its disposal as litter or in landfills [7].

Effective from December 31, 2022, in compliance with the Plastic Waste Management Rules, the thickness of plastic carry bags has been raised to one hundred and twenty microns [8]. Tamil Nadu was the fourth state to provide a ban on single-use plastic items as of January 1, 2019 [1]. The 3R concept, which emphasizes the importance of "Reduce, Reuse, and Recycle," can serve as a useful strategy to mitigate plastic hazards while also offering the opportunity to explore substitute materials [9].

A range of techniques, including physical, chemical, and biological methods, can accomplish the remediation of plastics [10]. Researchers have conducted various studies to investigate how plastic usage knowledge, attitude, and practice affect the environment [11-14]. They reported in these studies that most of the people had knowledge of the health hazards caused by inappropriate plastic waste disposal. However, there was a lack of awareness about the biodegradability of plastics among many individuals. With this background knowledge, we carried out the present study to estimate the prevalence and factors associated with plastic usage and recycling, as well as awareness regarding plastic hazards and legislation about plastic bans.

## Materials and methods

Study design and study duration

We conducted this analytical cross-sectional study for six months, spanning from March 2023 to August 2023.

Study population

The study involves the participation of consenting adults over the age of 18 from both urban and rural settings at a tertiary care hospital in the Perambalur district.

Inclusion criteria

Individuals who have surpassed the age of 18 and presently live within the field practice area of a tertiary care hospital.

Exclusion criteria

Participants in the selected households or shops who were not present despite three visits during the study period.

Sample size and sampling technique

According to Danasekaran et al. study, 35% of the participants used single-use plastics for food and grocery packing and storage [1]. Considering the above prevalence with a precision of 4% and a 95% confidence interval, we calculated the sample size using the formula, N = 3.84* p*q/d^2^. Hence, we needed a minimum sample size of 546 for the study in the rural area. By collecting 600 samples from both rural and urban areas, we have gained 1200 samples. An online random number generator was used to choose approximately 10 villages from the rural areas and 10 wards from the urban areas in the district of Perambalur based on the weight of the population from the 2011 Census. The research team randomly selected the streets for data collection in the chosen villages and wards. We included both commercial and residential areas in our selection. In commercial areas, we conducted a survey on a shop-by-shop basis, while for residential areas, we conducted a survey door-to-door. If the shop's door remained closed despite three visits during the survey, we surveyed the adjacent shop instead. We used the voter ID to verify that the participant was older than 18 years. Once verification was done, participants provided their consent, and the study proceeded through the use of a questionnaire.

Ethical clearance and informed consent

We received approval from the institutional ethics committee of Dhanalakshmi Srinivasan Medical College and Hospital before the start of our study (approval number: IECHS/IRCHS/No.319). We ensured that we fully informed study participants of the study's objectives, potential risks and benefits by obtaining their informed consent prior to conducting the study.

Data collection

The interviewer used the semi-structured questionnaire as a research tool for data collection. The questionnaire included socio-demographic information, such as age, gender, place of residence, level of education, and occupation. In the second section, data were collected on plastic usage, including frequency, influencing factors, and disposal methods. We included knowledge concerning the plastic ban and its necessities in the third section. The fourth section dealt with the examination of individuals' perceptions of BPA.

Statistical analysis

We entered the gathered data in Microsoft Excel (Microsoft Corporation, Redmond, WA) and analyzed it using SPSS trial version 26 (IBM Corp., Armonk, NY). The categorical variables were denoted as frequency and percentage. We represented the continuous variables using the mean and standard deviation. We used the chi-square test and Fischer's exact test, where applicable, to evaluate the association between plastic users and categorical variables. If the p-value was less than 0.05, we regarded it as statistically significant.

## Results

There were 1200 participants who took part in the study. The mean age of the samples who took part in our study was 44.46 ± 15.09 years. A significant proportion of the study participants, specifically 687 (57.3%), were female and 502 (41.8%) had completed their schooling. Approximately 446 (37.2%) of the study participants were engaged in agriculture. Of the study participants, 710 individuals (59.2%) selected a nuclear family type as their preference. We have described the socio-demographic details of the study participants in Table 1.

**Table 1:** Socio-demographic details of the study participants (n = 1200)

Variables		N (%)
Age in years		44.47 ± 15.09 (Mean ± SD)
Gender	Male	513 (42.8%)
	Female	687 (57.3%)
Education	Graduated	442 (36.8%)
	Completed Schooling	502 (41.8%)
	No formal education	256 (21.3%)
Residence	Rural	625 (52.1%)
	Urban	575 (47.9%)
Occupation	Agriculture	446 (37.2%)
	Non-Agriculturist	435 (36.3%)
	Unemployed	319 (26.6%)
Type of family	Joint family	360 (30%)
	Nuclear family	710 (59.2%)
	Three generation family	130 (10.8%)

**Figure 1:**
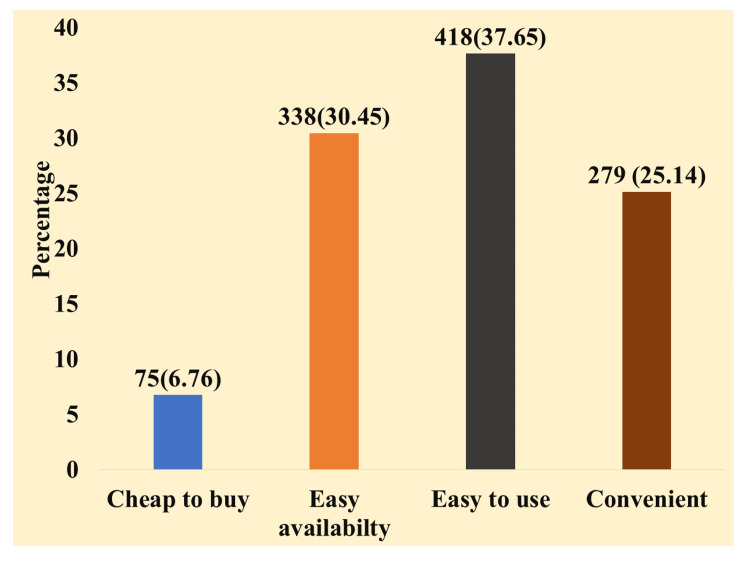
Factors influencing usage of plastic (n = 1110)

In this study, around 92.5% of the participants, namely 1110 out of 1200 individuals, confirmed the usage of plastic in their day-to-day routines. Therefore, we evaluated the determinants that impact the utilization of plastics among the participants. Of the factors considered, 418 (34.8%) respondents showed the ease of use as the reason for their use of plastic, followed by 338 (28.2%) who cited its availability, and 279 (23.3%) who mentioned its convenience. They considered plastics to be inexpensive to purchase, according to 76.3% of the respondents. We have described the factors influencing the usage of plastic in Figure 1.

**Figure 2:**
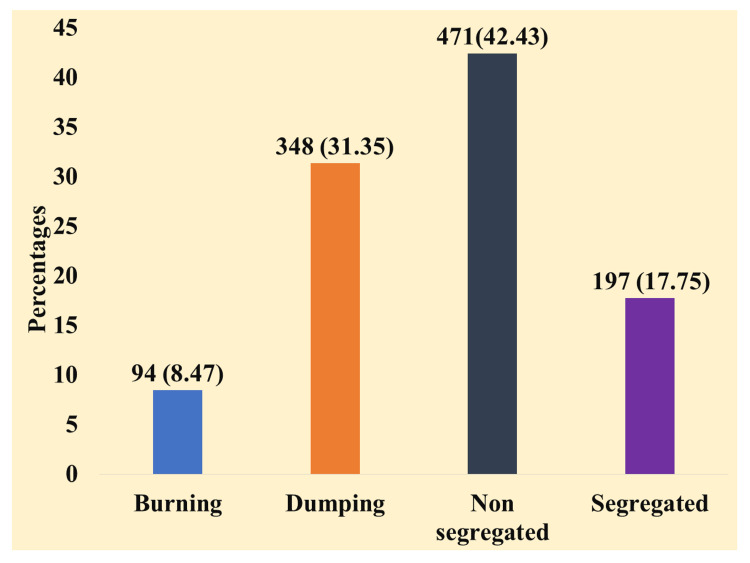
Methods of waste disposal (n = 1110)

Among the plastic users, about 418 (34.8%) of the people dispose of plastics directly in municipal vehicles or public dustbins without segregation of waste, followed by dumping (338 (28.2%)), which is the most unsanitary method of disposal of waste, and about 94 (7.8%) dispose of the waste by burning. About one-fourth of the plastic users, 197 (16.4%), dispose of the waste by proper segregation methods. We have described the methods of waste disposal among the study participants in Figure 2.

We conducted an evaluation to determine the extent to which individuals were aware of the term 'plastic ban,' with 91.9% (1103 individuals) stating their familiarity and 84.8% (1008 individuals) acknowledging their awareness of the plastic ban in Tamil Nadu. But, on further assessment, only 419 (34.9%) responded that they are aware of the plastic ban and regulation in India. Among the study participants, about 1013 (84.4%) agreed that a plastic ban is a necessity, about 115 (9.6%) disagreed with the necessity of a plastic ban, and about 72 (6%) of the participants replied neutrally to the necessity of a plastic ban. We have described the awareness of the plastic ban and its regulation in Table 2.

**Table 2:** Awareness of plastic ban and its regulation (n = 1200)

Variables		Frequency (%)
Awareness of plastic ban	Yes	1103 (91.9%)
	No	97 (8.1%)
Awareness of plastic ban in our state	Yes	1008 (84%)
	No	192 (16%)
Awareness of plastic laws and regulation in India	Yes	419 (34.9%)
	No	781 (65.1%)
Necessity of plastic ban	Agree	1013 (84.4%)
	Disagree	115 (9.6%)
	Neutral	72 (6%)

In the study, the researchers evaluated the participants' perceptions regarding BPA, and only 188 (15.7%) were aware of BPA. The study also evaluated the participants' perceptions of plastic recycling, and 610 (50.8%) were aware of it, while 1111 (92.6%) were aware of plastic alternatives such as paper, cloth, jute bags, etc. We have described the perception on BPA and plastic reuse in Table 3.

**Table 3:** Perception on bisphenol A (BPA) and plastic reuse (n = 1200)

Variables		n (%)
Awareness on bisphenol A (BPA)	Yes	188 (15.7%)
	No	1012 (84.3%)
Awareness of plastic recycling	Yes	610 (50.8%)
	No	495 (41.3%)
	Never heard	95 (7.9%)
Awareness of plastic alternatives	Yes	1111 (92.6%)
	No	89 (7.4%)

When considering socio-demographic characteristics such as age, gender, education, occupation, residence, and family type, our study found that the prevalence of plastic users was higher than non-users. The individuals belonging to the age group ≤ 40 years (99.3%) exhibit more frequent use of plastic than those in the age group > 40 years. These differences in proportion are statistically significant, with a p-value of 0.001. Our study discovered that the proportion of plastic usage among male participants (96.3%) was significantly higher than among female participants, with the difference being statistically significant (p-value 0.001). The study revealed that plastic usage was considerably higher amongst the graduates, with 99.3% (439 participants) using it, in contrast to the participants with no formal education, who had the least usage of only 78.5% (201 participants). The study findings showed a statistically significant difference in proportion between these groups, with a p-value of 0.001. According to our study, individuals living in urban areas use plastic more frequently than those in rural settings. The proportion of plastic usage among urban inhabitants is remarkably high, reaching 562 (97.7%), with a statistically significant difference in proportion, as shown by a p-value of 0.001. The non-agriculturist participants, comprising 98.9% of the study population, exhibited a greater tendency to use plastic compared to the agriculturist and unemployed participants. We observed a significant difference in proportions (p-value 0.001). The proportion of plastic users is significantly higher (95.6%) among participants belonging to joint families as compared to those in nuclear and three-generation families. The statistical analysis has confirmed the significance of this difference with a p-value of 0.001. We have described the association between basic characteristics and perception of plastic hazards in Table 4.

**Table 4:** Association between basic characteristics and perception of plastic hazards

Variables		Plastic users		p-value
		Users	Non-users	
Age in years	≤ 40	449 (99.3%)	3 (0.7%)	0.001
	41–59	486 (92%)	42 (8%)	
	≥ 60	175 (79.5%)	45 (20.5%)	
Gender	Male	494 (96.3%)	19 (3.7%)	0.001
	Female	616 (89.7%)	71 (10.3%)	
Education	Graduate	439 (99.3%)	3 (0.7%)	0.001
	Schooling	470 (93.6%)	32 (6.4%)	
	No formal education	201 (78.5%)	55 (21.5%)	
Residence	Rural	548 (87.7%)	77 (12.3%)	0.001
	Urban	562 (97.7%)	13 (2.3%)	
Occupation	Agriculturist	402 (90.1%)	44 (9.9%)	0.001
	Non-agriculturist	430 (98.9%)	5 (1.1%)	
	Unemployed	278 (87.1%)	41 (12.9%)	
Family type	Joint family	344 (95.6%)	16 (4.4%)	0.001
	Nuclear family	659 (92.8%)	61 (7.2%)	
	Three generation family	107 (82.3%)	23 (17.7%)	

## Discussion

We have analyzed the usage of plastics in our study. The study has showcased that all the socio-demographic characters taken in the study had a significant association with the plastic users.

Of the study participants, we found that 472 (39.3%) adhered to non-segregated waste disposal methods, whereas only 197 (16.4%) followed the segregation method, which is considered a better waste disposal technique. In 2017, Ranjeeta Kakoti conducted a study in India that showed that dustbins and public garbage bins are the major methods of waste disposal, followed by dumping, which is consistent with our research [15]. In Nepal, a study conducted by Ashish Khanal in 2022 among the youth population concluded that 80.1% of the participants practiced waste segregation [16]. The reason for this could be the strict laws in Nepal and the promotion of waste segregation.

Our research showed that plastic usage was more prevalent among younger participants. The results of a 2023 study conducted by Coco Chin et al. on Malaysians over the age of 18 suggest that those aged 46 and over possess a greater understanding of plastic pollution, while individuals aged 31 to 45 exhibit positive practices and attitudes [17]. We can attribute the reason for the similarity to the fact that people who are around 40 years old are more used to living with less plastic than the younger generation, who use plastic in almost everything.

Our study reveals that the usage of plastics was higher among males (96.3%, n=494) than females. In 2022, Ashish Khanal conducted a study among Nepal's youth population on single-use plastic, which determined that the gender and source segregation of waste were statistically significant. However, the study did not find any association between the two attributes [16]. Findings from a 2023 study by Hamza and Mahmoud, which surveyed adults in Egypt, showed that women held a positive attitude toward the utilization and hazards of plastic products [11]. Whereas a study conducted in the Philippines in 2021 by John Jamir Benzon R. Aruta among undergraduates concluded that males have an intention to reduce plastic use than females [18]. The difference may be because of the distinct geographic location and diverse perception, resulting in a disparity of opinion.

Participants with a higher level of education use plastic more frequently. Participants who receive higher levels of education use plastic less frequently, according to the research [19,20]. The study by Joseph et al. among adults in Mangalore in 2016 found that greater awareness of plastic hazards was observed among participants with higher education levels [3]. Although participants with higher education levels had greater awareness of plastic hazards, they still used plastic more frequently, which could explain the discrepancy.

Our study has revealed that the utilization of plastic is more prevalent among participants living in urban areas (562, or 97.7%) as opposed to those inhabiting rural areas. Similarly, a study conducted in the urban soil of Nanjing by Zhou et al. in 2023 on microplastics concluded that the main influencing factors of microplastics changed along with urbanization [21]. A study conducted in urban slums of Central Uganda by Mukama et al. in 2015 concluded that practices in waste disposal and separation were poor despite a high willingness to take part in initiatives to improve waste management [22].

Compared to the agriculturists and unemployed participants, we observed that the non-agriculturists were the most frequent users of plastic. According to Joseph et al.'s study in Mangalore among adults in 2016, semi-professionals and professionals have more awareness than other classes of occupation [3].

The results of our study show that joint-family participants showed a higher level of plastic usage than those from nuclear and three-generation families. Similarly, a study conducted in Chennai among adults by Manoj Raghavan in 2019 showed that people in nuclear families have a better awareness and attitude than those in joint families [23]. The explanation for this phenomenon could be the larger number of individuals in a joint family, which creates a conducive atmosphere for the transmission and dissemination of knowledge in a more efficient and suitable manner.

According to our study, less than one-third of the study participants had knowledge of BPA. In a study conducted in Bhopal by Priya et al. in 2016, among school students, only 9 (3%) of them knew about the leaching properties of plastics, and none of the participants knew about BPA [6]. Despite the outcomes being comparable, there is a significant fluctuation in similarity. Differences in population and investigation time may have contributed to this variation. When knowledge of BPA was minimal, during the earlier time frame, the similarity was lower.

Limitations

Our study has used multistage sampling, because of the constraints of limited resources. We could not extrapolate the findings to other regions of the country because the study only included participants from a specific geographic area. A multi-centric study with probability sampling technique all over the state in Tamil Nadu could yield better results. The assessment is lacking measures that could help determine the duration or severity of the situation. Since the study solely depends on the subjective responses of the participants, bias could affect the data arising from social desirability or selective recall bias. The association found in this study might not be causal since this was a cross-sectional study.

## Conclusions

Through our research, we have determined that the usage of plastic is more prevalent among study participants. The most common method of waste disposal is non-segregation of waste and dumping, which has to be reduced. In our study, higher the education, higher the usage of plastics, and urban dwellers are the higher users of plastics. Hence, awareness and implication of plastic hazards have to be increased. The younger generation is the major plastic users; hence, the importance of the hazards of plastic has to be strengthened. Awareness of plastic ban and legislation is present in a considerable number of people, whereas BPA awareness is present in only less than one-fifth of the study population. Most of the population was aware of plastic alternatives but still used plastic in their routine.
